# Foreign Matter in Alimentary Canal

**Published:** 1895-11

**Authors:** Judson Black

**Affiliations:** Richmond, Mich.


### FOREIGN MATTER IN ALIMENTARY CANAL.

BY JUDSON BLACK, V.S.,
RICHMOND, MICH.

A MARE, nine years of age, weighing about 900 pounds, came into present owner's hands on Friday, August 23, 1895. She was purchased from the owner of a grist-mill, and had run in an enclosure in which the mill was situated and had access to the sweepings of the mill. On Sunday following she was taken with an attack of diarrhœa, the passage consisting chiefly of unmasticated grain. I was called to see her Monday. The symptoms were those of colic, accompanied by a very depressed appearance. She would eat except when pain was very violent; bowels constipated; pulse very slightly accelerated at any time during sickness; temperature from 101° to 103° F.

Opiates were administered to relieve pain, ℥viij. aloetic bolus followed in twelve hours by ℥iv. sulp. mag. In twenty-four hours after ball no results nor any indications of peristalsis, gave 1½ pints linseed oil. In thirty-six hours from ball seeing no indications of getting any movement, I gave an intratracheal injection of eserine, gr. j.; pilocarpine gr., ss. This dose caused terrible agony for half an hour, when she passed a quantity of coarse sand and gravel in which I found pebble-stones as large as hazelnuts, several slivers of glass (the largest measuring three-fourths of an inch), pieces of zinc, stovepipe iron (one piece two inches long), and pieces of leather—about two pounds in all. The succeeding passages were composed of much the same material. After four very good evacuations the effects of the drug passed off. In twelve hours afterward I repeated the dose with results the same, excepting the last passage, which was comparatively free from foreign matter.

The bowels responded nicely to purgatives and case convalesced slowly, owing no doubt to lacerations in gastro-intestinal tract. She showed symptoms of pain after each meal for several days after bowels became natural in their movement.

